# How to create a faculty development program that transforms medical education according to actual institutional needs: evidence-based approach and experience at the University of Rijeka, Faculty of Medicine, Croatia

**DOI:** 10.3389/fmed.2025.1513119

**Published:** 2025-02-18

**Authors:** Nina Pereza, Goran Hauser, Sanja Dević Pavlić, Ivana Marić, Vlatka Sotošek, Tina Grgasović, Jasenka Mršić-Pelčić

**Affiliations:** ^1^Centre for Improving Teacher Competencies and Communication Skills, Faculty of Medicine, University of Rijeka, Rijeka, Croatia; ^2^Department of Medical Biology and Genetics, Faculty of Medicine, University of Rijeka, Rijeka, Croatia; ^3^Department of Internal Medicine, Faculty of Medicine, University of Rijeka, Rijeka, Croatia; ^4^Department of Internal Medicine, Clinical Hospital Centre Rijeka, Rijeka, Croatia; ^5^Department of Anatomy, Faculty of Medicine, University of Rijeka, Rijeka, Croatia; ^6^Department of Anesthesiology, Reanimatology, Emergency and Intensive Care Medicine, Faculty of Medicine, University of Rijeka, Rijeka, Croatia; ^7^Department of Clinical Medical Science II, Faculty of Health Studies, University of Rijeka, Rijeka, Croatia; ^8^Faculty of Medicine, University of Rijeka, Rijeka, Croatia; ^9^Department of Basic and Clinical Pharmacology and Toxicology, Faculty of Medicine, University of Rijeka, Rijeka, Croatia

**Keywords:** medical education, faculty development, flipped classroom, teacher competencies, evidence-based medicine, needs-based education, medical students

## Abstract

**Aim:**

Although previous studies demonstrated short, medium and long-term effectiveness of faculty development programs (FDP) for medical teachers, there is a lack of studies describing the methodology for creating a sustainable comprehensive FDP in medical education. We present the methodology for creating the “Modern and Practical Medical Education (MPME),” a comprehensive four-month educational FDP tailored to actual institutional needs, and the initial results of its implementation and transformation of medical education at the University of Rijeka, Faculty of Medicine, Croatia (EU).

**Materials and methods:**

We conducted a multi-phase mixed-method cross-sectional study from July 2021 to June 2024 consisting of three steps: (1) construction of MPME FDP basic structure according to local/national priorities/specificities, literature and expertise, (2) a 360-degree current state analysis for adaptation of basic structure to actual institutional needs, and (3) analysis of the FDP education effectiveness. Step 2 included syllabi analyses and group interviews with 65 teachers’ at 8 departments (qualitative), as well as the analysis of medical students’ perspective using the “Medical students’ questionnaire” on 236 participants (quantitative), and 23 interviews with 8 participants using the “Medical students’ focus group” (qualitative). Step 3 included the quantitative analysis of 40 medical teachers’ self-assessments for 30 teachers’ competencies before and after MPME education using the “MPME questionnaire,” and qualitative analysis of measurable program outcomes.

**Results:**

The MPME FDP consists of three major modules with seven courses, and is based on a tripartite flipped classroom model. The results of qualitative and quantitative 360-degree analysis identified common weak spots in teachers’ competencies, which were used for program development. Program effectiveness was demonstrated through a highly significant change in the self-assessment for all 30 teachers’ competencies before and after education (*p* < 0.001), and numerous measurable educational outcomes.

**Conclusion:**

The creation of a comprehensive and sustainable FDP in medical education should be based on a three-step quantitative and qualitative process that includes the 360-degree analysis of actual institutional needs, and the effectiveness of the program education. This methodology has a highly significant positive effect on teachers’ competencies at the individual level, and creation of educational projects that transform medical education at the institutional level.

## 1 Introduction

Faculty development (FD) is an organized strategy of a higher education institution (HEI) that is focused on professional advancement and growth of academicians by providing systematic and purposeful development of knowledge, skills and attitudes in the fundamental activities of institutions, including teaching, scientific research and administration (1). It has been described as having a decisive role in sustaining academic vitality, endorsing dignitary improvements and strategies that are executed in a professional manner (2, 3).

The existence of organized professional educational FD strategies at HEIs in the healthcare professions, especially medical education, is of the utmost importance for at least two equally crucial reasons. First, unlike many other studies in higher education, the medical studies curricula seldom include mandatory pedagogical education that would enable professional development of future medical doctors for becoming medical teachers (4, 5). Moreover, the rapid change in modern trends and advances in innovative teaching and learning methods in medical education, such as flipped classroom, interactive applications for case-based learning, virtual reality or clinical simulation, requires the formation of separate organizational units specifically dedicated to providing opportunities for professional educational FD, usually through various means of continuing medical education.

Unfortunately, although the FD system is extensively developed and deeply rooted in the foundational administrative structure of HEIs in medical education in the primarily English speaking countries, such as the United States of America, Canada, Australia and United Kingdom, there is a major deficiency of organized institutional support to professional educational development in the European Union, with few exceptions proving otherwise (3). The situation in the European Adriatic and Balkan countries is similar, with the University of Rijeka, Faculty of Medicine in the Republic of Croatia being one of the rare examples of a HEI that has its own FD organizational unit, i.e., the Center for Improving Teacher Competencies and Communication Skills since 2017 (6). The Center has been offering a wide range of FD programs (FDP) for educational development of medical teachers since its foundation. However, the COVID pandemics in 2020 and 2021 led to the spontaneous shutdown of existing onsite FDPs, which inevitably imposed the need to redesign the Center’s educational offer with a significant shift in FDPs content and methodology.

Creating a completely new and contemporary FDP according to actual institutional needs, which would simultaneously provide constructive support to medical teachers in developing and/or improving their competencies, and lead to a transformation and modernization of medical education is no simple task. The task does not become any easier when taking into account the concurrent national and regional specificities that reflect on the system of medical education in each country. For example, the Republic of Croatia is part of the European Union and, thus, part of the European Higher Education Area (EHEA), which directs the rules and regulations for the implementation of comparable and compatible education systems at the HEIs in Europe in accordance with the Bologna Process (7). In addition, the specificities of the healthcare system organization, the type of elementary and high school education, as well as particular cultural heritage of the Adriatic and Balkan area, directly influence the organization and implementation of the medical study, which is conducted as a “University Integrated Undergraduate and Graduate Study of Medicine.” All these factors clearly indicate that the rules and concepts for complementary teacher training, i.e., FD in medical education cannot simply be copied or outsourced from another system, especially from countries that are based on an entirely different organization of medical education at HEIs.

Furthermore, previous systematic reviews and meta-analyses emphasize that there is no universal FDP structure that would benefit all HEIs equally, but rather that it should correspond to actual institutional needs (3, 8). In addition, best practices indicate that a basic FDP should last at least 3 months and preferably be structured in a course format (3). However, to the best of our knowledge, there are no previous studies describing the methodology behind the creation of such a comprehensive and foundational institutional FDP for medical teachers, as well as subsequent testing of its effectiveness in implementation (8).

Therefore, the aim of this study is to present the methodology for creating and developing the “Modern and Practical Medical Education (MPME),” a comprehensive four-month educational FDP tailored to the actual institutional needs of medical teachers, as well as the initial results of its implementation and the transformation of medical education at the University of Rijeka, Faculty of Medicine, Croatia.

## 2 Materials and methods

**Figure 1:**
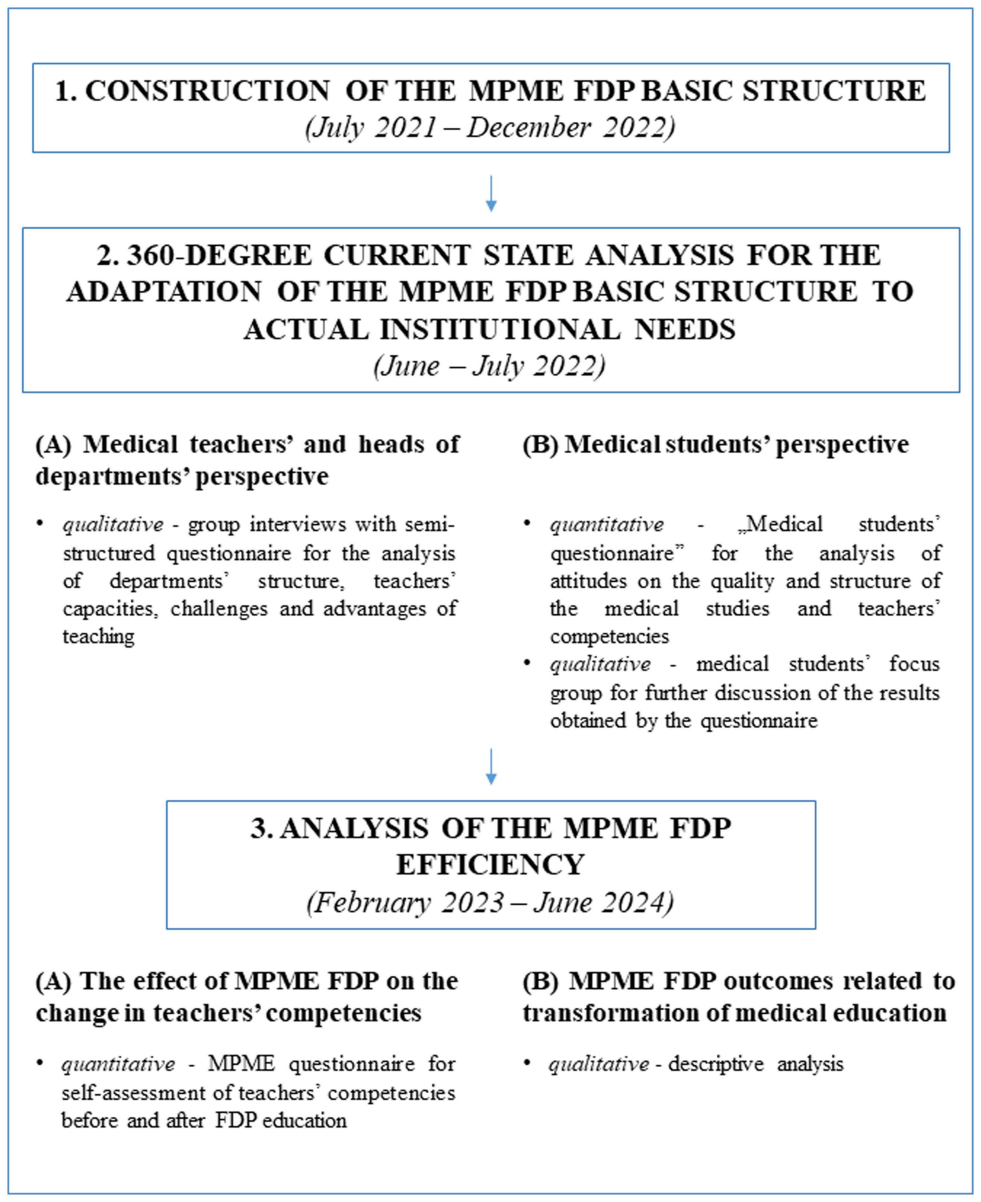
Flowchart representing the design of the study.

In order to obtain a comprehensive overview of the current needs of medical students and teachers necessary for the creation of the MPME FDP, as well as the effectiveness of the created program, we conducted a multi-phase mixed-method cross-sectional study that consisted of questionnaires (quantitative), descriptive analyses, group and focus groups interviews (qualitative). Considering the complex multistep process underlying the design of this study, the approach delineating each phase of the research with the corresponding methodology is presented as a Flowchart in Figure 1.

### 2.1 Construction of the MPME FDP basic structure

The MPME FDP basic structure was planned from July until December 2022 based on institutional, local and national priorities/specificities, personal expertise and literature. The original concept was created by the program coordinator (NP) and was envisioned as a comprehensive, four-month lifelong learning program divided into three major modules consisting of seven major courses (Table 1) (6). The major advantage of MPME is that the entire program is based on flipped classroom, which is an innovative approach for an FDP. The flipped classroom for every course in the MPME FDP is a tripartite system consisting of an introductory onsite session based on discussions about the challenges teachers encounter in relation to the topic of each course, which is followed by an online asynchronous course, and finalized with an onsite workshop for the application of the acquired principles in own practice. The asynchronous online courses last from 2 to 4 weeks depending on the course, and contain all theoretical principles in the form of “e-Contents for learning,” which consist of educative materials (short texts, recorded lectures and other original video contents), standardized forms and questions for repetition. The “e-Contents for learning” consist of >2000 documents and > 200 video materials.

**Table 1:** Structure of the MPME FDP.

Course	ECTS	Status
MODULE 1 – How is teaching created in medical education?		
1. Initial planning of teaching	2	M
2. Selecting learning methods, creating teaching materials and conducting lessons	2.5	M
3. Monitoring progress and assessing students and teaching	1.5	M
MODULE 2 – What are the specificities of teaching in medical education?		
1. Simulation of clinical teaching	2	M/E
2. Integration and (re)contextualization of basic courses through early exposure to clinical contents	2	M/E
MODULE 3 – How to modernize teaching in medical education?		
3. Application of e-learning tools in medical education	2	M
4. English as a medium of instruction in medicine	1.5	E
MODULE 4 – Advanced courses for medical teachers*		
5. Teacher training for conducting peer review in medical education*	1.5	E

ECTS, European Credit Transfer System; E, elective; M, mandatory; *Introduced in July 2024.

The program is intended for medical teachers who have at least 2 years of teaching experience in various healthcare professions studies, regardless of their own profession. The program is based on five interconnected principles, including personalized approach, individual mentoring, practical application of measurable products after the program, reflective practice and transformative learning. At the beginning of the program, each participant is paired with one mentor and an individual development plan is created based on their self-assessment of teachers’ competencies, expectations from the program, as well as the evaluation of one teaching unit by their choice. Throughout the program, each participant adapts their own existing teaching unit/course and creates new ones in the form of performance-based assessment. Finally, MPME is based on the system of triple reflection, i.e., every participant is continuously assessed by the mentor, other participants and themselves.

#### 2.1.1 Selection and training of MPME mentors

In addition to the MPME program coordinator/head mentor (NP), the coordinator selected an additional five MPME mentors at the University of Rijeka, Faculty of Medicine based on rigorous criteria for teaching excellence. These included previous training in medical education methodology, continuous excellent student evaluations, awards for teaching excellence, educational publishing activities, years of teaching experience and other variable criteria, such as number of coordinated courses etc. All five mentors were included in the first MPME cycle as participants and had to pass additional mentor training to be assigned with mentees from the second MPME cycle onwards.

### 2.2 360-degree current state analysis for the adaptation of the MPME FDP basic structure to actual institutional needs

After the construction of the MPME FDP basic structure, but before the creation of learning materials, a comprehensive, 360-degree current-state-analysis of the quality of teaching and teachers’ competencies, as well as the analysis of actual institutional needs was conducted at the University of Rijeka, Faculty of Medicine. The 360-degree analysis was conducted in June and July 2022, and included the analysis of: (A) medical teachers’ and heads of departments’ perspective, and (B) medical students’ perspective.

#### 2.2.1 Medical teachers’ and heads of departments’ perspective

The analysis of the medical teachers’ and heads of departments’ perspective was conducted as a qualitative analysis to analyze the structure and quality of the teaching process, as well as current state and needs of medical teachers in relation to the aims of the MPME FDP. We randomly selected 20% of faculty departments and, for each department, the analysis consisted of three parts, including a peer review of one teaching unit, detailed preparations for group interviews, and group interviews with department teachers and heads using a semi-structured questionnaire. Detailed preparations were conducted by three authors (NP, JMP, GH), which included the analysis of the list of courses with coordinators, course syllabi and results of anonymous student evaluations for every course and teacher in the last two academic years. NP moderated the interviews that lasted from 1 to 1.5 h, whereas JMP or GH were present as observers.

The semi-structured questionnaire consisted of two groups of questions (available on personal request). The first group referred to general information, including the number of department teachers, teachers’ professions, number of teachers with pedagogical education, opinions about their advantages and limitations in teachers’ competencies. The second group comprised six sub-groups of open-ended questions that correspond to MPME modules and courses, including “Planning of teaching” (20 questions), “Selecting learning methods, creating teaching materials and conducting lessons” (20 questions), “Monitoring progress and assessing students and teaching” (11 questions), “Clinical teaching” (10 questions), and “Pre-clinical teaching” (6 questions). From this predetermined set of 67 questions, a personalized questionnaire was made for each department depending on their specificities in education. During group interviews, questions were posed to the entire group.

#### 2.2.2 Medical students’ perspective

##### 2.2.2.1 Bipartite concept of study

The analysis of medical students’ perspective on the quality and structure of medical studies, as well as teachers’ competencies at the University of Rijeka, Faculty of Medicine included a research that consisted of two interconnected consecutive parts. The first part included the quantitative analysis of medical students’ attitudes through a specifically designed questionnaire for the afore-mentioned assessment, whereas the second part included the formation a medical students’ focus group for further discussion and qualitative analysis of the results obtained through the questionnaire. In addition, the results of both parts were converted to innovative educative materials that became integrated into various parts of the MPME FDP.

All students who participated in the study attended the University Integrated Undergraduate and Graduate Study of Medicine at the University of Rijeka, Faculty of Medicine.

##### 2.2.2.2 Part 1 – development and structure of the “medical students’ questionnaire” for the evaluation of attitudes on the quality of medical studies and teachers’ competencies

The questionnaire for the evaluation of medical students’ attitudes on the quality and structure of the medical studies and teachers’ competencies at the University of Rijeka, Faculty of Medicine, i.e., “Medical students’ questionnaire” was created in 2022 at the Center for Improving Teacher Competencies and Communication Skills based on expertise and institutional needs (Supplementary material 1).

The questionnaire is divided into two parts. The first part contains questions about medical students’ demographical data, such as the year of study and average grade. In the second part, the aim was to create a questionnaire that would complement the structure of the major modules and courses of the MPME FDP for easier implementation of results. Therefore, the “Medical students’ questionnaire” is divided into five groups of 55 questions. Group 1 (“Planning of teaching”) consists of 13 questions, group 2 (“Selecting learning methods, creating teaching materials and conducting lessons”) of 15 questions, group 3 (“Monitoring progress and assessing students and teaching”) of 6 questions, group 4 (“Clinical teaching”) of 13 questions, and group 5 (“Pre-clinical teaching”) of 8 questions. A total of 42 questions measure satisfaction, frequency and quality, and are rated on a five-point Likert-type scale with respective descriptions in which “1” always represents the lowest value, and “5” represents the highest value. The remaining five questions include ranking, six questions require a written answer, and two questions refer to selecting one answer.

The contents of the “Medical students’ questionnaire” differed for medical students in the pre-clinical (first, second, third) and clinical years (fourth, fifth and sixth). The questionnaire for the pre-clinical years consisted of 42 questions from groups 1–3 and 5, whereas the questionnaire for the clinical years contained all 55 questions from all groups.

##### 2.2.2.3 Distribution of the questionnaire to medical students

The final version of the “Medical students’ questionnaire” is a set of six online questionnaires created with the LimeSurvey GmbH program, versions 2.67.1 and 6.5.4 (Survey Services & Consulting, Hamburg, Germany) with separate links for each study year, i.e., from the first to the sixth year. The “Medical students’ questionnaire” was distributed to medical students of all 6 years of the University Integrated Undergraduate and Graduate Study of Medicine at the end of lecture period (June and July) in two different academic years, including in the academic year 2021/2022 for the first time, and in the academic year 2023/2024 for the second time. The questionnaires were distributed in association with the Student Council of the Faculty of Medicine, University of Rijeka, separately for each study year and each student filled only the questionnaire that referred to their current year of study. The distribution of the online questionnaire for each study year was conducted through two different channels, including official e-mail addresses, and internal student groups on mobile applications. The links to the questionnaires were sent three times in one-week intervals. Considering that this was an internal survey, the aim was to obtain a completion rate from at least 10 to 15% of medical students in each study year (9). All students participated in the research anonymously and voluntarily.

Only the results from the academic year 2021/2022 were used for creating the MPME FDP, whereas the results from the second round were used to check and confirm the previous results.

##### 2.2.2.4 Part 2 – medical students’ focus group

In order to better understand the medical students’ attitudes obtained through the “Medical students’ questionnaire” in the academic year 2021/2022, a “Medical students focus group” was created for further elaboration and discussion of the results. The focus group was established in October 2022 in cooperation with the Rijeka branch of “CroMSIC – Croatian Medical Students’ International Committee” and consisted of 8 medical students from the fourth, fifth and sixth years, who applied after an open call. All students participated in the research non-anonymously and voluntarily. The emphasis of focus groups was on group dynamics, unlike the group interviews with medical teachers and heads of departments, in which the focus was on participants answering questions without extensive interaction.

From October 2022 to April 2023, 4 sessions on 23 different topics were organized. For each topic, the medical students received a set of questions together with the corresponding results from the “Medical students’ questionnaire” at least 3 days in advance. In addition, the sessions were divided into two parts for each topic. The first part consisted of 20–30 min preparation and framework discussion between the interviewer (NP) and all students, followed by a maximum of 10-min video-recorded interview and discussion with a maximum of 2 representative students. The video recordings were converted into innovative learning material for teachers and integrated into the MPME FDP learning management system (LMS).

### 2.3 Analysis of the MPME FDP effectiveness

#### 2.3.1 Participants

The analysis of MPME FDP effectiveness was conducted as a retrospective quantitative and qualitative study, and included all medical teachers who enrolled and completed the first three cycles of the program, including the first cycle from February to June 2023, the second from October 2023 to February 2024, and the third from February to June 2024. The effectiveness of the program was evaluated using two different approaches, including quantitative analysis of the self-assessment of teachers’ competencies before and after education, as well as qualitative analysis of measurable program outcomes in terms of the effect on transforming medical education at the institutional level.

#### 2.3.2 The effect of MPME FDP on the change in teachers’ competencies

##### 2.3.2.1 Development and structure of the “MPME questionnaire” for self-assessment of teachers’ competencies before and after education

The effect of education provided by the MPME FDP on the change in teachers’ competencies was measured separately for each participant using the “Questionnaire for self-assessment of teacher competencies,” i.e., the “MPME questionnaire,” which was distributed to each participant twice, once at the beginning and once at the end of the program.

Considering that, in addition to the creation and implementation of such a comprehensive FDP, our primarily vision was to design a program that would be personalized and individualized according to the actual needs of each teacher, we needed to create or take over an appropriate existing questionnaire that could objectively measure the effect of the MPME FDP on each individual teacher. Unfortunately, because we could not find a suitable questionnaire in the existing literature, we aimed to develop a new one based on our expertise and using the data we obtained from the 360-degree needs-based analysis at the University of Rijeka, Faculty of Medicine.

The “MPME questionnaire” was designed in 2022 at the Center for Improving Teacher Competencies and Communication Skills, University of Rijeka, Faculty of Medicine to measure specific outcomes of the corresponding FDP (Supplementary material 2). The “MPME questionnaire” is divided into two parts. The first part contains questions for demographical data on teachers, such as department, gender, years of teaching experience, teaching position, list of coordinated courses, and courses in which they participate.

The second part of the “MPME questionnaire” is divided into five major groups of competencies that are precisely aligned with the major modules and courses of the MPME FDP, and the structure of the afore-mentioned questionnaire distributed to medical students. In addition, the competencies for self-assessment within each group precisely follow the major learning outcomes for each course. Group 1 (“Planning of teaching”) contains 9 competencies, group 2 (“Selecting learning methods, creating teaching materials and conducting lessons”) 8 competencies, group 3 (“Monitoring progress and assessing students and teaching”) 4 competencies, and group 5 (“Application of e-learning tools in medical education”) 3 competencies. Finally, group 4 is divided into two subgroups, one that contains 6 competencies for “Clinical teaching” (4A) and one that contains 4 competencies for testing “Awareness of pre-clinical teachers toward clinical teaching concepts” (4B), and each participant filled only one of these two subgroups depending on their professional position. The group 4B was not assessed after the MPME FDP education. Finally, it is important to emphasize that the “MPME questionnaire” contains competencies that are complimentary to medical students’ attitudes present in their questionnaire to allow a comparison between the teachers’ and medical students’ perspectives on the same topics.

Collectively, the “MPME questionnaire” consists of 30 competencies for clinical teachers and 28 competencies for pre-clinical teachers, and each competency is rated on a five-point Likert-type scale: 1 – very low level of competence, 2 – low level of competence, 3 – average level of competence, 4 – moderately high level of competence, and 5 – high level of competence. In addition, for each competency, there is the option to select “NA” if the competency is not applicable in participant’s practice or if they cannot assess their level of competency.

##### 2.3.2.2 Questionnaire distribution and data collection

The questionnaire was available to participants online on the MPME LMS as a Word document, which had to be downloaded, completed and uploaded on the same LMS. Completion of the questionnaire was non-anonymous and mandatory to start and complete the program. The non-anonymous results of the “MPME questionnaire” were accessible only to the individual teacher, MPME FDP coordinator and the teacher’s mentor.

For the purpose of this retrospective study, the MPME FDP coordinator and first author (NP) anonymized and de-identified the questionnaires by assigning each teacher a separate non-identifying code for further statistical analysis (10). The cross-linking of teachers to the codes was known and accessible only to the first author (NP).

#### 2.3.3 MPME FDP outcomes related to transformation of medical education at the institutional level

MPME FDP outcomes related to transformation of medical education at the institutional level were analyzed as short-term and long-term effects.

Considering that the MPME FDP is based on performance-based assessment, the short-term effects referred to a descriptive analysis of the products developed by teachers throughout the program. These measurable program outcomes included teaching plans for one existing and one new teaching unit, PowerPoint presentation for a mini-lecture, different forms of questions for various types of assessment, a clinical skills or clinical reasoning project, a project for early clinical exposure, and for course coordinators a revised course syllabus.

The analysis of long-term effects also included a descriptive analysis of the informal feedback received from teachers who completed the MPME FDP regarding additional changes they might have implemented in their practice (e.g., accreditation of new elective courses, implementation of case-based learning, etc.).

### 2.4 Ethical approval

All relevant bodies at the University of Rijeka and the Faculty of Medicine in Rijeka approved this research. The Ethics Committee for Biomedical Research of the University of Rijeka, Faculty of Medicine approved the evaluation of medical students’ attitudes on the quality of medical studies and teachers’ competencies (Class: 007–08/24–01/44, Order Number: 2170-1-42-04-3/1-24-5). In addition, the Senate of the University of Rijeka approved the accreditation of the MPME FDP with the self-assessment of teachers’ competencies as part of the mandatory evaluation within the program (Class: 644-07/22-01/47, Order Number: 2170-57-12-22-8). Considering that this part of the research was a retrospective study, no additional ethical approvals were needed.

### 2.5 Data analysis

#### 2.5.1 Quantitative analysis – statistical analysis

Statistical analysis was conducted in the Statistica program, version 14.0.0.15 (StatSoft, Inc., Tulsa, OK, USA). Nominal variables are shown in absolute and relative frequencies. The normality of the distribution of numerical variables was examined by the Kolmogorov–Smirnov test. For a more rigorous interpretation of the results, the level of statistical significance was set at *p* < 0.01 for all analyses.

##### 2.5.1.1 Analysis of medical students’ attitudes on the quality of medical studies and teachers’ competencies

All numerical variables are presented by median and interquartile range (IQR), except for grade average, which is presented by median and range. Differences in the total number of participants between the academic years 2021/2022 and 2023/2024 were calculated using the Chi-square test, whereas differences in grade averages between different study years of the two academic years were calculated using the Mann–Whitney test. Differences in medians of attitudes between the pre-clinical and clinical years of study (first + second + third vs. fourth + fifth + sixth year) were calculated by the Mann–Whitney test for independent samples, whereas differences between individual years of study were calculated by the Kruskal–Wallis test and post-hoc analysis. In addition, the Friedman test was used for questions that required ranking.

##### 2.5.1.2 Analysis of the self-assessment of teachers’ competencies before and after MPME FDP education

All numerical variables are presented by median and IQR, except for years of teaching experience and number of courses in which teachers participate, which are presented as median and range. Differences in medians of grades for each competency before and after MPME FDP education was calculated using the Wilcoxon Matched Pairs Test.

#### 2.5.2 Qualitative analysis

##### 2.5.2.1 Analysis of medical teachers’ and heads of departments’ perspective

The qualitative analysis of course syllabi in the preparation activities for group interviews included the analysis of the adherence of their structure to the standardized syllabus of the University of Rijeka, Faculty of Medicine, as well as existence of constructive alignment, and adherence of each syllabus component to medical education methodology.

Group interviews with medical teachers and heads of departments were not audio nor video recorded, but the same scorer was present at all sessions, and made all the transcripts verbatim in an anonymized form. After every session, the scorer and the interviewer (NP) arranged all data thematically into 5 predetermined major categories (General impression, Teachers’ opinion on their competencies, Examples of best practices, Objective needs for improvement, and Infrastructural challenges), and analyzed them using further coding to obtain subcategories of answers. The codes and interpretation were compared and discussed by the interviewer (NP) and one observer (JMP). Considering the high, almost complete interrater reliability, eventual minor disagreements were discussed until consensus was reached.

##### 2.5.2.2 Medical students’ focus group and creation of innovative educative materials for the MPME FDP

The 23 focus group interviews with medical students were video recorded. The obtained data was not further transcribed nor coded but was rather converted into video learning materials for teachers and integrated into the MPME LMS into corresponding modules and courses.

##### 2.5.2.3 MPME FDP outcomes related to transformation of medical education at the institutional level

The MPME FDP short-term and long-term outcomes related to transformation of medical education at the institutional level included a descriptive analysis of the frequency of created products applicable to actual teaching practice.

## 3 Results

### 3.1 360-degree current state analysis for the adaptation of the MPME FDP basic structure to actual institutional needs

#### 3.1.1 Medical teachers’ and heads of departments’ perspective

Medical teachers’ and heads of departments’ analysis included 20% of randomly selected departments at the University of Rijeka, Faculty of Medicine (8/39), with 3 pre-clinical (37.5%) and 5 clinical departments (62.5%). We conducted 8 peer reviews (data not shown), analyzed 16 course syllabi, and conducted 8 separate groups interviews at different departments with a total of 57 teachers and 8 heads of departments.

The key results of course syllabi analyses at different departments included general deficiencies and non-compliance with the standardized institutional form for course syllabus, as well as issues in the formulation of course aims, learning outcomes, contents, assessment, and consequently lack of constructive alignment. All results point to non-adherence of the syllabus as a crucial document for planning of teaching with the basic rules of medical education methodology.

In addition, the results of group interviews were categorized into five major categories as indicated previously. The “General impression” (Category 1) of teachers and heads of departments about the current-state analysis and construction of the MPME FDP was positive, and all participants were cooperative and willing to participate in group interviews. However, specific differences were noted between pre-clinical and clinical teachers regarding the mode of communication about personal limitations in teachers’ competencies and willingness to attend an FDP. Clinical teachers were more open and motivated toward attending an FDP in medical education, considering it an opportunity for professional growth and excellence. On the other hand, pre-clinical teachers were more “protective” toward their teaching process and potential room for personal improvement. In Category 2, “Teachers’ opinions on their competencies” varied from department to department. In general, pre-clinical and clinical teachers could not list at least five teacher competencies in medical education, with most of them mentioned only “presentation skills” and “interacting with students.” Additionally, when asked which competencies they would like to improve, the answer was usually absent. Considering that group interviews were conducted in positive surroundings, different improvements in the teaching process were arranged for each department, including the replacement of the concept of seminars in which students hold PowerPoint presentations with introduction of case-based learning, recording the performance of clinical skills that students rarely see during practicals, etc. Furthermore, course syllabi analyses and group interviews led to the recognition of best practices at each department, all of which were integrated into the MPME FDP (Category 3 – “Examples of best practices”). In Category 4, common objective needs for improvement were noted with substantial differences between pre-clinical and clinical courses. In general, pre-clinical courses require modernization, primarily in terms of introducing active learning methods and flipped classroom, student-centered learning, early integration of clinical contents, and reduction of information overload. In clinical courses, we identified the necessity for standardizing the teaching and learning of clinical skills and reducing non-uniformity between teachers, as well as introducing objective exams and interactive case-based learning. All teachers would highly benefit from education in writing learning outcomes, giving feedback, raising awareness about teachers’ competencies and thinking about teaching from students’ perspective. Finally, although the MPME FDP cannot assist in improving “Infrastructural challenges of teachers” (Category 5), it was important to learn about challenges not associated with teachers’ competencies. Most clinical teachers indicate “the lack of time and teaching personnel” as the biggest issue in clinical teaching.

#### 3.1.2 Medical students’ perspective – quantitative analysis of the “Medical students’ questionnaire”

##### 3.1.2.1 Participants

Table 2 presents the number of medical students by study year who completed the “Medical students’ questionnaire” in the academic years 2021/2022 and 2023/2024, as well as their average grades. The target response rate of at least 10% was achieved for all study years in both academic years, with an overall response rate of 15.2%. In total, the questionnaire was completed by 236 medical students from all 6 years in both academic years, of which 112 (47.5%) were in their pre-clinical years of study, and 124 (52.5%) in their clinical years of study. There were no statistically significant differences in the number of participants or their average grade between the two academic years (*p* = 0.454 and 0.669, respectively).

**Table 2:** Number of medical students by study year who filled the “Medical students’ questionnaire.”

Academic year 2021/2022				Academic year 2023/2024			
Year of study	Number of students, (N)	Response rate, (%)	Average grade, median (range)	Year of study	Number of students, (N)	Response rate, (%)	Average grade, median (Range)
1	18	13.3	4.55 (4.00–4.90)	1	20	14.9	4.43 (3.80–4.80)
2	27	20.0	4.33 (4.00–4.90)	2	22	16.1	4.23 (3.40–4.60)
3	12	10.0	4.22 (4.00–4.68)	3	13	10.4	4.40 (3.90–4.70)
4	24	18.2	4.00 (3.48–4.40)	4	15	11.4	4.50 (4.00–5.00)
5	29	20.6	4.40 (3.80–4.90)	5	16	13.8	4.35 (3.83–4.59)
6	26	22.0	4.35 (3.80–4.80)	6	14	11.1	4.30 (3.55–4.64)
Total	136	17.2	4.30 (3.48–4.90)	Total	100	13.1	4.31 (3.40–5.00)
*p*-value^a^					0.453		
*p*-value^b^					0.669		

a Chi-square test for testing differences in the number of participants between academic years 2021/2022 and 2023/2024.

b Mann–Whitney test for testing differences in total average grades between academic years 2021/2022 and 2023/2024.

Considering that there were no statistically significant results between the two academic years when individual questions were analyzed in the “Medical students’ questionnaire” (data not shown due to extensiveness), the results of both academic years were analyzed and presented jointly.

##### 3.1.2.2 Analysis of results in group 1 questions (“planning of teaching”)

The results for questions in group 1 (“Planning of teaching”) that required rating on a scale from 1 to 5 are shown in Table 3. In the combined analysis of all 6 years, a median grade of 3 was determined for all questions that referred to the current state analysis of the quality of syllabi, alignment of courses’ aims with learning outcomes and contents, representation of authentical medical education, mutual content complementation between lectures, seminars and practicals, as well as differences in methodology between different forms of teaching (questions 2–5, 7–10). However, when the median grades for the same questions were further compared between pre-clinical and clinical years, the differences reached high statistical significance (*p* < 0.001), with the median for the clinical years always being one grade lower.

**Table 3:** Results for questions in group 1 (“Planning of teaching”) in the “Medical students’ questionnaire.”

Question Rate on the scale from 1 to 5	All 6 years combined (*N* = 236)	Preclinical years (*N* = 112)	Clinical years (*N* = 124)	*p*-value*
	Median (IQR)	Median (IQR)	Median (IQR)	
1. How important is the syllabus to you to successfully navigate a course?	5 (4–5)	5 (4–5)	5 (3–5)	NS
2. Evaluate the quality of current syllabi.				
Clarity	4 (3–4)	4 (3–5)	3 (2–4)	<0.001
Structuredness	4 (3–5)	5 (4–5)	4 (2–4)	<0.001
Availability of all relevant information	3 (2–4)	4 (3–5)	3 (2–4)	<0.001
3. How much are aims and learning outcomes of courses aligned with their contents?	3 (2–4)	4 (3–4)	3 (2–3)	<0.001
4. How much are aims, learning outcomes and contents of courses aligned with the actual needs of medical students for their future profession?	3 (2–3)	3 (2–4)	3 (2–3)	<0.001
5. Do teachers explain the aims and learning outcomes at the beginning of each class?	3 (1–4)	3 (2–4)	2 (1–3)	<0.001
6. How important is it to you that the contents of lectures, seminars and practicals complement each other meaningfully?	5 (5–5)	5 (5–5)	5 (4–5)	NS
7. How much does the content of lectures, seminars and practicals complement each other currently?	3 (2–4)	4 (3–4)	3 (2–3)	<0.001
8. How much does the concept of lectures and seminars differ in methodology currently?	3 (2–3)	3 (2–4)	3 (2–3)	0.018
9. Do you think teachers hold organized preparations for classes?	3 (2–4)	4 (2–5)	2 (1–3)	<0.001
10. How much are ECTS credits aligned with the actual needs of courses?	3 (2–3)	3 (2–4)	2 (1–3)	<0.001

ECTS, European Credit Transfer System; IQR, interquartile range; NS, not significant; *Mann–Whitney test.

Another important analysis included the comparison between questions 1 and 2, as well as questions 6 and 7. The difference in the median grades between question 1 (“How important is the syllabus to you to successfully navigate a course”) and 2 (“Evaluate the quality of current syllabi”) was also statistically significant (*p* < 0.001). Similarly, a statistically significant difference (*p* < 0.001) was observed in the median grades between question 6 (“How important is it that the contents of lectures, seminars and practicals complement each other meaningfully?”) and question 7 (“How much does the content of lectures, seminars and practicals complement each other currently?”).

In addition, the analysis of questions that required ranking, showed the following results. In response to the question “Which parts of the syllabus do you study before the beginning of a course?” (question 11), the students answered in the following order of importance: assessment (Mean Rank 2.43), schedule (Mean Rank 2.75), learning methods (Mean Rank 2.86), contents (Mean Rank 3.43), learning outcomes (Mean Rank 4.65), course aims (Mean Rank 4.88). Also, the ranking for question 12, “On which part of the syllabus do you form an impression about the course before its start?,” showed that assessment was in the first place (Mean Rank 3.17), followed by course contents (Mean Rank 3.21), learning methods (Mean Rank 3.34), schedule (Mean Rank 3.70), learning outcomes (Mean Rank 3.72), and aims (Mean Rank 3.86). Finally, in response to the question “According to which component do you think lectures, seminars and exercises in all courses should differ?” (question 13), most students selected the learning methods (144/236, 61.1%), followed by aims and learning outcomes (72/236, 30.5%), and contents (70/236, 29.7%), whereas 40/236 (16.9%) of medical students thought that they should not differ.

##### 3.1.2.3 Analysis of results in group 2 questions (“Selecting learning methods and conducting lessons”)

The results for questions in group 2 (“Selecting learning methods and conducting lessons”) that required rating on a scale from 1 to 5 are shown in Table 4. Similar to the results of group 1, for those questions in which differences between median grades reached statistical significance, the medians were mostly one grade lower for clinical years in comparison to pre-clinical years.

**Table 4:** Results for questions in group 2 (“Selecting learning methods and conducting lessons”) in the “Medical students’ questionnaire.”

Question Rate on the scale from 1 to 5	All 6 years combined (*N* = 209)	Preclinical years (*N* = 97)	Clinical years (*N* = 112)	*p*-value*
	Median (IQR)	Median (IQR)	Median (IQR)	
1. How much are teachers prepared for their classes?	4 (3–5)	4 (3–5)	4 (3–4)	<0.001
2. If different teachers teach the same teaching unit on a certain course, how uniform are they in achieving the learning outcomes and scope of the compulsory content?	3 (2–4)	3 (2–4)	3 (2–4)	0.003
3. If different teachers teach the same teaching unit on a certain course, how uniform are they in their instructional material (e.g., presentation)?	3 (2–4)	3 (1–4)	3 (2–4)	NS
4. How satisfied are you with the current state of receiving feedback on your progress during seminars and practicals?	3 (1–3)	3 (1–3)	2 (1–3)	0.002
5. Evaluate the current level of interactivity at different forms of teaching.				
Lectures	2 (1–3)	2 (1–3)	2 (1–2)	NS
Seminars	3 (2–4)	4 (3–4)	3 (2–4)	0.014
Practicals	4 (3–5)	4 (3–5)	4 (3–4)	0.002
6. Evaluate the current level of individualization at different forms of teaching.				
Lectures	1 (1–2)	1 (1–2)	1 (1–2)	NS
Seminars	2 (1–3)	3 (1–3)	2 (1–3)	0.013
Practicals	3 (1–4)	3 (1–4)	3 (2–4)	NS
7. Evaluate the current level of relevance of the teaching content for the future profession of students.				
Lectures	3 (2–4)	3 (2–4)	3 (2–3)	0.003
Seminars	3 (2–4)	4 (2–4)	3 (2–4)	0.004
Practicals	4 (3–5)	4 (3–5)	4 (3–5)	NS
8. How important is the quality of compulsory literature to you?				
Recency	5 (3–5)	4 (3–5)	5 (4–5)	NS
Relevance	5 (4–5)	5 (4–5)	5 (4–5)	NS
Availability	5 (4–5)	5 (4–5)	5 (4–5)	NS
9. Evaluate the current quality of compulsory literature.	3 (2–4)	4 (3–4)	3 (2–4)	<0.001
10. How important is a good learning environment to you?	5 (4–5)	5 (4–5)	5 (4–5)	NS
11. How much do teachers encourage the establishment of your identity as a future healthcare professional?	3 (2–4)	4 (3–4)	3 (2–4)	<0.001
12. How much do teachers encourage the establishment of your identity as a future medical educator?	3 (1–3)	3 (2–4)	2 (1–3)	<0.001

IQR, interquartile range; NS, not significant; *Mann–Whitney test.

Some of the more distinctive results refer to questions 2 and 3, regarding the uniformity between different teachers in the same courses “in achieving the learning outcomes and scope of the compulsory content” and “in their instructional material,” which reached the median grades of only 3 (IQR from 2 to 4) for both questions on all years of study.

Furthermore, questions 4–7 evaluated the current representation of the FAIR principles in the medical studies, i.e., Feedback, Activity, Individualization and Relevance in different forms of teaching (11). For example, the median grade for medical students’ satisfaction with the current state of “receiving feedback on their progress in seminars and practicals” (question 4) was 3 (IQR from 1 to 3) for all 6 years combined, and only 2 (IQR from 1 to 3) for students in their clinical years. When asked to choose a reason for their discontent with the current state of receiving feedback, most students of all 6 years agreed that “feedback was not given often enough” (142/209, 67.9%), followed by “feedback is not individualized” (100/209, 47.8%), and “feedback is given in a negative way” (63/209, 30.1%). For questions 5–7, which referred to the representation of interactivity, individualization and relevance in different forms of teaching, the lowest median grades were always detected for lectures in all 6 years combined, as well as in pre-clinical and clinical years. Finally, when students were asked to “Determine the order of relevance of the four components of FAIR principles” (question 14), “relevance of contents for future profession” was determined as the most important (Mean Rank 1.90), followed by “class individualization” (Mean Rank 2.23), “active participation in classes” (Mean Rank 2.50), and “receiving feedback on progress” (Mean Rank 3.36).

The following two questions, 8 and 9, tested the difference between the perception on “the importance of compulsory literature to medical students” and the “current state of compulsory literature,” which was statistically significant (median grades 5 vs. 3, *p* < 0.001).

Medical students were also asked to “Determine the order of 15 characteristics of a successful teacher during class,” and the top three most important characteristics were: “preparedness” (Mean Rank: 5.48), “enthusiasm” (Mean Rank: 6.18) and “expertise” (Mean Rank: 6.61), whereas “wit” (Mean Rank: 12.16) was considered as the least important.

In the final question in group 2, medical students were given the opportunity to write their answers to the question “List your own challenges in creating a successful relationship with teachers during class.” A staggering 163/209 students wrote their answers (78%), and the primary challenge was determined to be “low level of prior knowledge” (54/163, 33.1%), followed by “fear of authority” (42/163, 25.8%), lack of communication with students (15/163, 9.2%), and other sporadic reasons, such as student demeaning, information overload, etc.

##### 3.1.2.4 Analysis of results in group 3 questions (“Monitoring progress and assessing students and teaching”)

The results for questions in group 3 (“Monitoring progress and assessing students and teaching”) that required rating on a scale from 1 to 5 are shown in Table 5. The median grades for all four questions in all 6 years combined, as well as pre-clinical and clinical years were low, especially for questions 3 and 4 regarding the “uniformity of different teachers in evaluation criteria” and “appreciation of students’ comments in anonymous course evaluations,” both of which had a median grade of 2. In addition, similar to questions in group 1 and 2, median grades in clinical years were statistically significantly lower than in pre-clinical years.

**Table 5:** Results for questions in group 3 (“Monitoring progress and assessing students and teaching”) in the “Medical students’ questionnaire.”

Question Rate on the scale from 1 to 5	All 6 years combined (*N* = 177)	Preclinical years (*N* = 85)	Clinical years (*N* = 92)	*p*-value*
	Median (IQR)	Median (IQR)	Median (IQR)	
1. How much is the assessment system aligned with the credit load of courses?	3 (2–4)	3 (2–4)	3 (2–3)	<0.001
2. How much is the content at different forms of assessment consistent with the material covered in class?	3 (2–4)	4 (3–4)	3 (2–3)	<0.001
3. How much are teachers uniform in the evaluation criteria for forms of assessment in which multiple teachers participate?	2 (1–3)	2 (1–3)	2 (1–2)	<0.001
4. How much do you think that teachers respect students’ comments in anonymous course evaluations?	2 (1–3)	3 (1–4)	2 (1–3)	0.007

IQR, interquartile range; NS, not significant; *Mann–Whitney test.

Furthermore, medical students were asked whether “the evaluation results are a reflection of the performance of students or teachers” (question 5) and 133/177 (75.2%) of students agreed that both were true. The final question in this group referred to the opinion of whether “students need to have all the required literature available for different forms of assessment” (question 6) and 137/177 (77.4%) of students agreed this was the case.

##### 3.1.2.5 Analysis of results in group 4 questions (“Clinical teaching”)

The results for questions in group 4 (“Clinical teaching”) that required rating on a scale from 1 to 5 are shown in Table 6. A total of 84 students in their fourth, fifth and sixth years of study completed this group of questions.

**Table 6:** Results for questions in group 4 (“Clinical teaching”) in the “Medical students’ questionnaire.”

Question Rate on the scale from 1 to 5	Clinical years (*N* = 84)	
	Median (IQR)	*p*-value*
1. Do teachers conduct student orientation on the first day of practical work (e.g., introduction to the clinic, staff, etc.)?	2 (1–4)	NS
2. Do teachers assess the different levels of clinical skills competencies in students at the beginning of courses?	1 (1–2)	NS
3. How much are different teachers uniform in teaching clinical skills in the same course?	2 (1–3)	NS
4. How much do different teachers have uniform instructions for patient presentation in the same course?	3 (2–3)	NS
5. How consistent are different teachers in providing feedback to students about their progress in mastering clinical skills in the same course?	2 (1–3)	NS
6. Evaluate the compatibility of your level of competence for individual clinical skills in the clinical skills booklet and reality.	2 (1–3)	<0.001**
7. How much are the contents of clinical courses too detailed for students, i.e., too specialized?	4 (3–4)	NS
8. Evaluate the importance of case-based learning.	5 (4–5)	NS
9. Evaluate the current level of representation of case-based learning in class.	3 (2–3)	NS
10. Evaluate the importance of using clinical reasoning in class.	5 (4–5)	NS
11. Evaluate the current level of representation of clinical reasoning in class.	2 (2–3)	NS

IQR, interquartile range; NS, not significant; *Kruskal-Wallis test between the fourth, fifth and sixth years of study; 4th vs. 5th and 4th vs. 6th year; 4th year (median 3), 5th year (median 2), 6th year (median 2).

The median grades were low for all questions that evaluated the current state of clinical teaching (questions 1–6, 7, 9, 11), with none of them reaching a median of 4. The lowest median grade of 1 was obtained for question 2 “Do teachers check the different levels of clinical skills competencies in students at the beginning of courses?” (IQR from 1 to 2). A median grade of 2 was obtained for student orientation (question 1), uniformity in teaching clinical skills (question 2), uniformity in giving feedback (question 5), compatibility of the competence level for individual clinical skills in the clinical skills booklet and reality (question 6), as well as the current level of representation of clinical reasoning in class (question 11).

There were no statistically significant differences in median grades between the different study years for any question except question 6 (“Evaluate the compatibility of your level of competence for individual clinical skills in the clinical skills booklet and reality”). In this question, fourth-year medical students gave a higher median grade than fifth- and sixth-year students (*p* < 0.001).

In addition, a statistically significant difference was obtained between medical students’ expectations on “the importance of case-based learning” (question 8) and “the importance of using clinical reasoning in class” (question 10) vs. their current representation in clinical teaching (question 9 and 11) (*p* < 0.001).

Finally, the last two questions were essay questions and referred to listing the challenges and advantages of clinical teaching. Again, an astounding 68 out of 84 medical students (81.0%) answered both questions. Most students (35/68, 51.5%) indicate “lack of learning clinical skills and/or lack of systematic learning of clinical skills and/or lack of uniformity of teachers in teaching clinical skills” as the major challenge, followed by “teachers not being interested for students” (20/68, 29.4%) and “teachers’ lack of time” (6/68, 8.8%). The results for major advantages of clinical teaching are not indicated here as they refer to individual courses.

##### 3.1.2.6 Analysis of results in group 5 questions (“Pre-clinical teaching”)

The final group of questions in the “Medical students’ questionnaire” referred to “Pre-clinical teaching” and the results for questions that required rating on a scale from 1 to 5 are shown in Table 7. Some of the more prominent results include those for question 3–5, all of which obtained a median grade of 4 (“How much is the scope of basic courses too detailed for students, i.e., too scientific?,” “Evaluate the importance of science in clinical teaching,” and “Are there differences in the quality of teaching by teachers who are the same and different professions than the future profession of students?”). Again, medical students in their clinical years gave lower grades than students in their pre-clinical years, which reached statistical significance for three out of five questions.

**Table 7:** Results for questions in group 5 (“Pre-clinical teaching”) in the “Medical students’ questionnaire.”

Question Rate on the scale from 1 to 5	All 6 years combined (*N* = 169)	Preclinical years (*N* = 83)	Clinical years (*N* = 86)	*p*-value*
	Median (IQR)	Median (IQR)	Median (IQR)	
1. Do the teachers conduct student orientation on the first day of practical work (e.g., introduction to the department, staff, etc.)?	3 (1–4)	3 (2–4)	2 (1–4)	0.022
2. How much do teachers associate the importance of basic content with clinical practice?	3 (2–4)	3 (2–4)	3 (2–3)	0.006
3. How much is the scope of basic courses too detailed for students, i.e., too scientific?	4 (3–4)	4 (2–4)	4 (3–4)	NS
4. Evaluate the importance of science in clinical teaching.	4 (2–4)	4 (3–4)	3 (3–4)	0.005
5. Are there differences in the quality of teaching by teachers who are the same and different professions than the future profession of students?	4 (2–4)	3 (2–5)	4 (3–4)	NS

Finally, the last questions were essay questions and referred to listing the challenges and advantages of pre-clinical teaching and 145 out of 169 students wrote an answer (85.8%). All of the comments mention information overload and lack of association of basic topics with clinical topics as the major challenges.

#### 3.1.3 Medical students’ perspective – qualitative analysis of the “Medical students’ focus group”

The 23 focus group interviews with medical students that were video recorded were converted into two innovative video learning material for teachers and integrated into the MPME FDP LMS into corresponding modules and courses. We called the first innovative format “Meducast,” which is a set of 18 short vidcasts (<10 min), in which students comment on the quality of the teaching process and teachers’ competencies based on the results from the “Medical students’ questionnaire.” The second format was called “MedXperience,” a set of 5 short highlights (<5 min) in which one medical student emphasizes a major message regarding their own positive or negative experiences or examples of good practice during medical studies. The complete list of Meducast and MedXperience formats are available as Supplementary material 3.

### 3.2 Analysis of the MPME FDP effectiveness

#### 3.2.1 Participants of the MPME FDP

A total of 44 medical teachers enrolled in the MPME FDP in the first three cycles of the program, including the first cycle from February to June 2023, second from October 2023 to February 2024, and third from February to June 2024. 42 teachers (95.5%) successfully completed the program, whereas one teacher dropped out due to termination of employment, and one teacher temporarily paused attending the program due to maternity leave. 12 teachers took part in the program in both the first and second cycle and 20 teachers in the third cycle.

Of the 42 medical teachers who successfully completed the MPME FDP, 27 were clinical teachers (64.3%) and the remaining 15 were pre-clinical teachers (35.7%). Most participants were employed at the Center for Proteomics (5/42, 11.9%), followed by the Department of Internal Medicine (4/42, 9.5%) and Department of Pediatrics (4/42, 9.5%).

A total of 34 participants were women (81.0%) and 8 were men (19.0%). According to their teaching position, 20 teachers were senior assistants/post-doctoral fellows (47.6%), 12 assistant professors (28.6%), 8 assistants (16.7%), and 3 tenured professors (7.1%), with no associate professors. 12 teachers were the coordinators of at least one course (28.6%). The median number of years of teaching experience was 9 (range 3–24), whereas the median number of courses in which teachers participated was 5 (range 1–15).

At the time of enrolment, completion of the MPME FDP was a mandatory condition for promotion to the teaching position of associate professor for 8/42 teachers in the period of 2 years (16.7%), 13/42 teachers in the period of three or more years (30.9%), whereas to as much as 22/42 (52.4%) participants, the completion of the program was not a mandatory condition for promotion.

The average grade of satisfaction with the program after three cycles is 4.8.

Of the 42 teachers who completed the MPME FDP, 40 teachers filled the “MPME questionnaire” for the self-assessment of teachers’ competencies before and after education (95.2%).

#### 3.2.2 The effect of MPME FDP on the change in teachers’ competencies

##### 3.2.2.1 Teachers’ competencies in groups 1–3 and 5

Table 8 presents the results of teachers’ self-assessment for group 1–3 and 5 competencies before and after MPME FDP education, which were the same for both clinical and pre-clinical teachers. Collectively, a statistically significant difference in the level of competency before and after MPME FDP education was determined for all 24 teachers’ competencies in all four groups of competencies, with the *p*-value <0.001. After the MPME FDP education, the median increased for two grades for most competencies (18/24, 75.0%), followed by one grade for two competencies (8.3%), 1.5 grades for two competencies (8.3%), and three and 2.5 grades for one competency each (8.3%). The highest increase, that of three grades occurred for the competency “Choosing an assessment method according to learning outcomes and methods,” which rose from 2 (1–3) to 5 (4–5), whereas the lowest increase, for one grade, occurred for competencies “establishing interaction with students during classes” and “using presentation skills,” which rose from 4 (4–4) / (3–4) to 5 (5–5) / 5 (5–5), respectively.

**Table 8:** Results of teachers’ self-assessment for group 1–3 and 5 competencies before and after MPME FDP education.

Group of competency	Before education		After education		*p*-value*
Group 1. Planning of teaching	N_V_	Median (IQR)	N_V_	Median (IQR)	
Evaluate your level of competency for:					
Creating a syllabus	30	3 (1–3)	39	5 (4–5)	<0.001
Creating a lesson plan	33	3 (1–3)	40	5 (4–5)	<0.001
Conducting authentical medical education (based on actual needs for the future profession of students)	34	3 (2–4)	40	4.5 (4–5)	<0.001
Knowing and conducting the roles of a course coordinator	34	3 (2–4)	39	5 (4–5)	<0.001
Knowing and conducting the roles of a teaching associate	36	3 (2–4)	40	5 (4–5)	<0.001
Designing and writing aims of lessons/courses	36	3 (1.5–3)	40	5 (4–5)	<0.001
Designing and writing learning outcomes of lessons/courses	40	2.5 (1–3)	40	5 (4–5)	<0.001
Designing and writing content of lessons/courses	40	3 (1.5–3)	40	5 (4–5)	<0.001
Choosing the form of teaching according to learning outcomes of teaching units	40	3 (1–4)	40	5 (4–5)	<0.001
Group 2. Selecting learning methods, creating teaching materials and conducting lessons					
Giving feedback to students according to structured models	36	3 (2–4)	40	5 (4–5)	<0.001
Establishing interaction with students during classes	39	4 (4–4)	40	5 (5–5)	<0.001
Choosing a learning method according to learning outcomes of teaching units	38	3 (2–4)	40	5 (4–5)	<0.001
Using authentic methods of passive learning in medical education	32	3 (2–3.5)	40	5 (4–5)	<0.001
Using authentic methods of active learning in medical education	33	3 (2–4)	40	5 (4–5)	<0.001
Using presentation skills	38	4 (3–4)	40	5 (5–5)	<0.001
Using facilitation skills	33	3 (2–3)	40	4.5 (4–5)	<0.001
Creating own teaching materials	39	3 (3–4)	40	5 (4–5)	<0.001
Group 3. Monitoring progress and assessing students and teaching					
Aligning the assessment system with the credit load of a course	32	2 (1–3)	40	4 (4–5)	<0.001
Choosing an assessment method according to learning outcomes and methods	32	2 (1–3)	40	5 (4–5)	<0.001
Creating different methods of written assessment (e.g., MCQ)	35	3 (2–4)	40	5 (4–5)	<0.001
Regular conduction of student evaluation of teaching	35	3 (1–4)	40	5 (4–5)	<0.001
Group 5. Application of e-learning tools in medical education					
Creating and administrating e-courses in the Moodle LMS	34	2 (1–4)	40	4 (4–5)	<0.001
Choosing and creating interactive contents in the Moodle LMS	34	2 (1–5)	40	4 (4–5)	<0.001
Monitoring and assessing students’ work in virtual environment	35	2 (1–3)	40	4 (3–5)	<0.001

IQR, interquartile range; LMS, learning management system; N_V_, number of valid responses (without answers “not applicable”); *Wilcoxon Matched Pairs Test.

Before and after education, the lowest median grades were observed in group 5 competencies (“Application of e-learning tools in medical education”) with a median grade of 2 and 4, respectively. In addition, the highest median before education was observed in group 2 competencies for “establishing interaction with students during classes” and “using presentation skills,” the two of which also had the lowest increase in median grades after education.

##### 3.2.2.2 Clinical teachers’ competencies in group 4A

Table 9 presents the results of teachers’ self-assessment for group 4 competencies before and after MPME FDP education, which were completed by clinical teachers only. Similar to groups 1–3 and 5, a statistically significant difference in the level of competency before and after MPME FDP education was determined for all 6 teachers’ competencies, with the *p*-value <0.001. After the MPME FDP education, the median increased for two grades for all but two competencies (4/6, 66.6%), whereas the remaining two increased for one grade (“teaching clinical skills” and “choosing an assessment method for the evaluation of clinical competence according to learning outcomes and learning methods”). Before education, the highest median grade was observed for the competency “teaching clinical skills” (4), whereas the remaining competencies were graded with 3.

**Table 9:** Results of clinical teachers’ self-assessment for group 4A competencies before and after MPME FDP education.

Group of competency	Before education		After education		*p*-value*
Group 4. Clinical teaching	N_V_	Median (IQR)	N_V_	Median (IQR)	
Evaluate your level of competency for:					
Teaching clinical reasoning	25	4 (3–4)	26	5 (4–5)	<0.001
Orienting students	23	3 (3–4)	26	5 (4–5)	<0.001
Using educational strategies for teaching in different clinical environments (e.g., one-minute preceptor)	22	3 (3–4)	26	5 (4–5)	<0.001
Teaching clinical skills	22	3 (3–4)	25	5 (4–5)	<0.001
Teaching in clinical simulation	24	3 (3–4)	26	5 (4–5)	<0.001
Choosing an assessment method for the evaluation of clinical competence according to learning outcomes and learning methods	21	3 (2–3)	26	4 (4–5)	<0.001

IQR, interquartile range; N_V_, number of valid responses (without answers “not applicable”); *Wilcoxon Matched Pairs Test.

##### 3.2.2.3 Pre-clinical teachers’ awareness of clinical concepts (group 4B)

The group 4B consisted of four questions that tested the pre-clinical teachers’ awareness of clinical concepts before education, including “teaching clinical reasoning,” “orienting students,” “applying early integration of clinical content into pre-clinical teaching” and “knowing educational strategies for teaching in authentic settings for the future profession of students (e.g., teaching in clinical environment).” The medians with IQRs for the four competencies were 2.5 (1–3), 2 (1–3), 1 (1–3) and 1 (1–3), respectively, with high numbers of teachers who selected the option “not applicable” (6/14 or 42.9%, 1/14 or 7.1%, 3/14 or 21.4% and 9/14 or 64.3%), respectively. After education, all teachers were familiar with the concepts.

#### 3.2.3 MPME FDP outcomes related to transformation of medical education at the institutional level

##### 3.2.3.1 Short-term outcomes

All teachers who completed the MPME FDP successfully revised and created teaching plans for one existing and one new teaching unit, respectively, with aims, learning outcomes, contents and assessment written according to the rules of medical education methodology, and constructively aligned the afore-mentioned components. In addition, all teachers created PowerPoint presentations for a mini-lecture, different forms of questions for various types of assessment according to best practices, created clinical skills or clinical reasoning project, a project for early clinical exposure, and course coordinators revised their course syllabi.

##### 3.2.3.2 Long-term outcomes

All long-term outcomes refer to fundamental transformation of medical education in individual courses or even entire departments. For example, 12 teachers who were or became course coordinators after completing the MPME FDP, completely transformed their courses in teaching and learning methodology and/or assessment. In addition, 1 new elective course was accredited and 2 are currently being created, 2 departments introduced the practice of professional meetings for teaching at the departmental level, whereas 1 major pre-clinical and 1 clinical course introduced case-based learning into seminars. However, the major and most important long-term outcome of all is currently being conducted at the Department of Internal Medicine as the “InterMed project,” which includes the virtual standardization of teaching and learning of clinical skills according to the principles of flipped classroom. Following the positive example of this project, three additional departments opted for a complete reform of the teaching and learning process with the creation of completely new educational materials. Finally, digital transformation of the assessment of clinical competences is currently being introduced at the institutional level, which primarily includes the creation of the objective structured clinical exams in an online application.

## 4 Discussion

### 4.1 The methodology behind the creation of an efficient FDP in medical education

In the present study, we elaborated on the methodology behind the creation of a comprehensive FDP in medical education, and presented the results of the effectiveness of the education provided for medical teachers at the University of Rijeka, Faculty of Medicine from January 2023 to July 2024. It is important to emphasize that the creation of the MPME FDP was one of the largest and most demanding educational projects at the Faculty, considering its complex and sophisticated infrastructure, with a large number of collaborators included in its creation. In addition, throughout the process, all of our activities were based on transparency, honesty and cooperation with medical students, teachers and administration at the institutional level. Unfortunately, considering that the concept of faculty development is less known in the European Union, and professional development of teachers is not on a desirable level of appreciation in the Republic of Croatia, such a wide approach toward creating a positive environment was of the utmost importance. Therefore, despite the implementation of an extensive evidence-based methodology, we are skeptical that the entire project would have been such a success without the continuous promotion of the positive atmosphere and necessity toward teacher education and its benefits.

Although all previous studies that analyzed the effectiveness of FDPs in medical education demonstrated effectiveness in the short, medium and long-term for medical teachers, they point to difficulties in creating innovative and sustainable educational programs (3). In addition, there is a general lack of studies describing the methodology for creating effective FDPs in medical education. Our study demonstrated both the effectiveness and sustainability of the MPME FDP, which stems from the three-year long creation and adaptation of the program according to the actual needs of medical students, leadership and medical teachers at the Faculty. Based on our results, we suggest that the methodology for creating a FDP in medical education should be based on three steps: (1) construction of the basic structure according to local and national priorities/specificities, literature and expertise, (2) a 360-degree current state analysis for the adaptation of the basic structure to actual institutional needs, and (3) analysis of the FDP effectiveness on the change in teachers’ competencies and transformation of the educational process in general. Steps 2 and 3 should include both quantitative and qualitative research for a better understanding and complementation of data. In addition, step 2 should include medical students, administration and medical teachers at the institutional level, whereas step 3 should include the same medical teachers attending the FDP before and after education. Of course, it is important to emphasize that although teachers’ self-assessments provide insights, they may not accurately reflect competence improvements due to biases or subjectivity but nevertheless give insight to teachers’ level of confidence and motivation for further professional development.

### 4.2 How did we eventually adapt the FDP to actual needs? Comparative compatibility between medical students and teachers regarding the current state of medical education and teachers’ competencies

Most of the results from the quantitative and qualitative research in Step 2 of our study, i.e., the 360-degree current state analysis of the medical studies and teachers’ competencies from the perspective of medical students, medical teachers and the administration were compatible and complemented each other in meaningful ways, which was crucial for content enrichment of the program.

In the quantitative part of the research, when we compared the results of the “Medical students’ questionnaire” and the “MPME questionnaire,” it became obvious that medical teachers at the University of Rijeka, Faculty of Medicine were aware of their own challenges in conducting medical education, allowing us to create meaningful and targeted learning materials based on “weak spots.” For example, medical students graded the “quality of current syllabi,” “presence of authentical medical education,” “meaningful complementation of lectures, seminars and practicals,” and “giving feedback” with a median grade of 3, the same grade with which teachers assessed their level of competence for “creating a syllabus,” “conducting authentical medical education,” “choosing the form of teaching according to learning outcomes of teaching units,” and “giving feedback according to structured models.” In addition, some of the results obtained from medical students were further emphasized when medical teachers’ assessed their level of competence. These mostly referred to assessment, especially “aligning the assessment system with the credit load of a course,” “choosing an assessment method according to learning outcomes and methods,” for which the median grade in teachers was one point lower than in students. Furthermore, a minority of results were opposite and these were quite interesting. For instance, medical students assessed the “current level of interactivity at different forms of teaching” with median grades 2, 3 and 4 for lectures, seminars and practicals, whereas medical teachers assessed their level of competence for “establishing interaction with students during classes” with the median grade of 4. The qualitative part of our research showed that medical students and medical teachers perceived the meaning of “interactivity” in completely different ways, i.e., medical students implied this concept to be “using active-learning strategies that facilitate the learning process,” while medical teachers believed this notion might refer to “basic communication skills with students.” Another interesting result refers to teaching skills in the clinical setting. Medical students graded both the “uniformity of teachers in teaching clinical skills,” and the “current level of representation of clinical reasoning in class” with a median of 2, whereas medical teachers believed they teach “clinical skills” with a median grade of 3, and “clinical reasoning” with a median grade of 4. The reason behind this discrepancy of results becomes more obvious from the qualitative part of the research, and the possible explanation might be that the clinical teachers equated their own clinical competence of conducting clinical skills and clinical reasoning with the manner in which they teach these skills to medical students. In other words, the importance of FD in medical education is already evident from these two results, which emphasize the need to teach teachers how to teach from the students’ perspective and use a reflective approach to teaching (12). These two results gave us the idea to implement the principles of transformative learning into specific parts of the MPME FDP.

The qualitative analysis of our research also revealed interesting conclusions for medical students, medical teachers and administration. For example, group interviews with medical teachers and heads of departments exposed certain differences between pre-clinical and clinical teaching. Clinical teachers demonstrated a more positive attitude toward FD from the beginning, which was opposite to initial expectations, and ultimately reflected in the higher ratio of clinical to pre-clinical teachers who enrolled and completed the MPME FDP. Considering that the quality of medical education was graded statistically significantly lower for clinical than pre-clinical teaching for most questions in the “Medical students’ questionnaire,” qualitative research showed that clinical teachers are definitely aware of this fact and, above all, are willing to commit to their professional development as medical teachers. Finally, the qualitative research involving medical students and the formation of the “Medical students’ focus group” allowed us to create two innovative learning formats for the MPME FDP, the Meducast and MedXperience video materials, both of which aim toward developing the affective learning domain in teachers.

In conclusion, all of the results obtained in the 360-degree analysis of the current state were used for the creation of different innovative learning materials for MPME, selection of appropriate learning methods, and adaptation of the teaching approach toward teachers of different professions and working positions.

### 4.3 Finally, how effective is the MPME FDP?

To answer this question, we measured the effectiveness of the MPME FDP in four different ways. First, considering that the program lasts 4 months, one of our biggest apprehensions was the attrition rate. However, after the first three cycles of the program, the attrition rate was 2.3% (1/44 due to termination of employment). Second, the effectiveness was measured as the change in self-assessment of teachers’ competencies, and the median grade for all 30 competencies evaluated before and after education increased significantly (*p* < 0.001) for one, two or even three grades. Third, the effectiveness was evaluated through objective and measurable program outcomes, in terms of actual products created by medical teachers for their own teaching practice, as well as projects that started the transformation of medical education at the institutional level. Finally, the effectiveness was measured through participant satisfaction with the program, which reached a mean of 4.8 after the first three cycles.

### 4.4 Advantages and limitations of the study

The major advantage of this study is the meticulous evidence-based methodology that included both quantitative and qualitative research in a three-step process for the creation and testing of effectiveness of a comprehensive FDP in medical education. In addition, the creation of the MPME FDP encompassed the perspectives of all relevant stakeholders of medical education, including medical students, medical teachers and administration, and integrated the needs and values of local, regional, national and European specificities into the program. The special value of the MPME FDP lies in the fact that more clinical teachers attended the program than pre-clinical teachers, which is a strength of its own. Finally, the large-scale projects that aim to transform the process of learning and teaching in medical education at the University of Rijeka, Faculty of Medicine, such as the “InterMed project” for introducing flipped classroom for the mastery of clinical skills.

One of the potential limitations of this study is the fact that the impact of the long-term effects on the transformation of medical education have just started and will require many years in advance for follow-up. For example, in the following period, we intend to incorporate external evaluations (e.g., peer review or student performance metrics) to triangulate findings from self-assessments. Similarly, it is important to continuously support medical teachers who finished the MPME FDP and encourage them in their future professional development through creation of new, less comprehensive FDPs, as well as individualized support where needed.

## 5 Conclusion

In the present study, we described the methodology behind the creation of a comprehensive four-month-long FDP in medical education, and presented the results of its effectiveness for medical teachers who attended the program at the University of Rijeka, Faculty of Medicine from January 2023 to July 2024.

We suggest that the methodology for creating a FDP in medical education should be based on three steps: (1) construction of the basic structure according to local and national priorities/specificities, literature and expertise, (2) a quantitative and qualitative 360-degree current state analysis for the adaptation of the basic structure to actual institutional needs, as well as (3) quantitative and qualitative analysis of the FDP effectiveness on the change in teachers’ competencies and transformation of the educational process in general. All activities must be based on transparency and cooperation with medical students, teachers and the administration to create a positive environment for lifelong learning. Finally, our results confirmed that this methodology has a highly significant effect on the change of 30 teachers’ competencies for teaching and learning in medical education (*p* < 0.001), and creation of educative projects that transform medical education at the institutional level. Perhaps the best conclusion of this study regarding the creation of comprehensive FDPs in medical education would be that “one size does not fit all” and that their creation requires “sophisticated tailoring of a suit that fits each individual separately.”

## Data Availability

The raw data supporting the conclusions of this article will be made available by the authors, without undue reservation.

## References

[1] Hamilton GC Brown JE. Faculty development: what is faculty development? Acad Emerg Med. (2003) 10:1334–6. doi: 10.1197/S1069-6563(03)00549-9 14644785

[2] Bilal GSY Chen S. The impact and effectiveness of faculty development program in fostering the faculty's knowledge, skills, and professional competence: a systematic review and meta-analysis. Saudi J Biol Sci. (2019) 26:688–97. doi: 10.1016/j.sjbs.2017.10.024, PMID: 31048993 PMC6486500

[3] Cotta RMM de Souza Ferreira E de Aguiar Franco F da Costa Souza Barros G Januário JPT Moreira TR. The effectiveness of faculty development programs for training university professors in the health area: a systematic review and meta-analysis. BMC Med Educ. (2024) 24:768. doi: 10.1186/s12909-024-05735-1, PMID: 39014385 PMC11253325

[4] O'Byrne GA. Are doctors born teachers? Br Med J (Clin Res Ed). (1988) 296:838–9. doi: 10.1136/bmj.296.6625.838 PMC2545117 3130936

[5] Stull MJ Duvivier RJ. Teaching physicians to teach: the underappreciated path to improving patient outcomes. Acad Med. (2017) 92:432–3. doi: 10.1097/ACM.0000000000001613, PMID: 28350605

[6] Pereza N Mršić-Pelčić J. Faculty educational development at the Faculty of Medicine in Rijeka. Medicina Fluminensis. (2023) 59:371–9. doi: 10.21860/medflum2023_309421

[7] European Ministers of Education. The Bologna declaration of 19 June 1999: Joint declaration of the European ministers of education. Bologna: The European Higher Education Area (1999).

[8] Steinert Y Mann K Centeno A Dolmans D Spencer J Gelula M. A systematic review of faculty development initiatives designed to improve teaching effectiveness in medical education: BEME guide no. 8. Med Teach. (2006) 28:497–526. doi: 10.1080/01421590600902976 17074699

[9] Chung L. What is a good survey response rate for online customer surveys? (2013). Available at: https://delighted.com/blog/average-survey-response-rate (Accessed October 17, 2024).

[10] Audette LM Hammond MS Rochester NK. Methodological issues with coding participants in anonymous psychological longitudinal studies. Educ Psychol Meas. (2020) 80:163–85. doi: 10.1177/0013164419843576, PMID: 31933497 PMC6943988

[11] Harden RM Laidlaw JM. Be FAIR to students: four principles that lead to more effective learning. Med Teach. (2013) 35:27–31. doi: 10.3109/0142159X.2012.732717 23121246

[12] Ramani S Leinster S. AMEE guide no. 34: teaching in the clinical environment. Med Teach. (2008) 30:347–64. doi: 10.1080/01421590802061613, PMID: 18569655

