# Supplementary material for: How to create a faculty development program that transforms medical education according to actual institutional needs: evidence-based approach and experience at the University of Rijeka, Faculty of Medicine, Croatia
Source: Front Med (Lausanne). 2025 Feb 18;12:1513119. doi: 10.3389/fmed.2025.1513119 (PMC11876177; doi:10.3389/fmed.2025.1513119)
Supplement: Supplementary file 1 [file Table_1.DOCX]

Supplementary Material 1

Supplementary table 1. The “Medical students’ questionnaire”

| **GROUP 1 – PLANNING OF TEACHING** | **ANSWER** |
| --- | --- |
| 1. How important is the syllabus to you to successfully navigate a course? | Rate on the scale from 1 to 5. |
| 2. Evaluate the quality of current syllabi.   - clarity, structuredness, availability of all relevant information |  |
| 3. How much are aims and learning outcomes of courses aligned with their contents? |  |
| 4. How much are aims, learning outcomes and contents of courses aligned with the actual needs of medical students for their future profession? |  |
| 5. Do teachers explain the aims and learning outcomes at the beginning of each class? |  |
| 6. How important is it that the contents of lectures, seminars and practicals complement each other meaningfully? |  |
| 7. How much does the content of lectures, seminars and practicals complement each other currently? |  |
| 8. How much does the concept of lectures and seminars differ in methodology currently? |  |
| 9. Do you think teachers have organized preparations for classes? |  |
| 10. How much are ECTS credits aligned with the actual needs of courses? |  |
| 11. Which parts of the syllabus do you study before the beginning of a course?   - course aims, course contents, course learning aims, learning methods, schedule, student assessment | Rank in the ascending order of importance. |
| 12. On which part of the syllabus do you form an impression about the course before its start?   - course aims, course contents, course learning aims, learning methods, schedule, student assessment |  |
| 13. According to which component do you think lectures, seminars and exercises in all courses should differ?   - aims and learning outcomes, contents, learning methods, they should not differ |  |

| **GROUP 2 - SELECTING LEARNING METHODS AND CONDUCTING LESSONS** | **ANSWER** |
| --- | --- |
| 1. How much are teachers prepared for their classes? | Rate on the scale from 1 to 5. |
| 2. If different teachers teach the same teaching unit on a certain course, how uniform are they in achieving the learning outcomes and scope of the compulsory content? |  |
| 3. If different teachers teach the same teaching unit on a certain course, how uniform are they in their instructional material (e.g. presentation)? |  |
| 4. How satisfied are you with the current state of receiving feedback on your progress during seminars and practicals? |  |
| 5. Evaluate the current level of interactivity at different forms of teaching.   - lectures, seminars, practicals |  |
| 6. Evaluate the current level of individualization at different forms of teaching.   - lectures, seminars, practicals |  |
| 7. Evaluate the current level of relevance of the teaching content for the future profession of students.   - lectures, seminars, practicals |  |
| 8. How important is the quality of compulsory literature to you?   - recency, relevance, availability |  |
| 9. Evaluate the current quality of compulsory literature. |  |
| 10. How important is a good learning environment to you? |  |
| 11. How much do teachers encourage the establishment of your identity as a future healthcare professional? |  |
| 12. How much do teachers encourage the establishment of your identity as a future medical educator? |  |
| 13. Determine the order of relevance of the following components for successful teaching (FAIR principles)   - active involvement in classes, class individualization, giving feedback on student progress, relevance of class contents for future profession | Rank in the ascending order of importance. |
| 14. Determine the order of characteristics of a successful teacher during class.   - availability, consistency, dedication to students, encouraging activity, enthusiasm, expertise, interest, justice, pandering, patience, preparedness, professionalism, respect, strictness, wit |  |
| 15. List your own challenges in creating a successful relationship with teachers during class. | Write an answer. |

| **GROUP 3 - MONITORING PROGRESS AND ASSESSING STUDENTS AND TEACHING** | **ANSWER** |
| --- | --- |
| 1. How much is the assessment system aligned with the credit load of courses? | Rate on the scale from 1 to 5. |
| 2. How much is the content at different forms of assessment consistent with the material covered in class? |  |
| 3. How much are teachers uniform in the evaluation criteria for forms of assessment in which multiple teachers participate? |  |
| 4. How much do you think that teachers respect students' comments in anonymous course evaluations? |  |
| 5. Are the evaluation results a reflection of the performance of students or teachers?   - students, teachers, both | Select one answer. |
| 6. Do you think students need to have all the required literature available for different forms of assessment?   - yes, no |  |

| **GROUP 4 - CLINICAL TEACHING** | **ANSWER** |
| --- | --- |
| 1. Do teachers conduct student orientation on the first day of practical work (e.g. introduction to the clinic, staff, etc.)? | Rate on the scale from 1 to 5. |
| 2. Do teachers check the different levels of clinical skills competencies in students at the beginning of courses? |  |
| 3. Are different teachers uniform in teaching clinical skills in the same course? |  |
| 4. Do different teachers have uniform instructions for patient presentation in the same course? |  |
| 5. How consistent are different teachers in providing feedback to students about their progress in mastering clinical skills in the same course? |  |
| 6. Evaluate the compatibility of your level of competence for individual clinical skills in the clinical skills booklet and reality. |  |
| 7. How much are the contents of clinical courses too detailed for students, i.e. too specialized? |  |
| 8. Evaluate the importance of case-based learning. |  |
| 9. Evaluate the current level of representation of case-based learning in class. |  |
| 10. Evaluate the importance of using clinical reasoning in class. |  |
| 11. Evaluate the current level of representation of clinical reasoning in class. |  |
| 12. What are the challenges of clinical teaching? | Write an answer. |
| 13. What are the advantages of clinical teaching? |  |

| **GROUP 5 – PRE-CLINICAL TEACHING** | **ANSWER** |
| --- | --- |
| 1. Do the teachers conduct student orientation on the first day of practical work (e.g. introduction to the department, staff, etc.)? | Rate on the scale from 1 to 5. |
| 2. How much do teachers associate the importance of basic content with clinical practice. |  |
| 3. How much is the scope of basic courses too detailed for students, i.e. too scientific? |  |
| 4. Evaluate the importance of science in clinical teaching. |  |
| 5. Are there differences in the quality of teaching by teachers who are the same and different professions than the future profession of students? |  |
| 6. What are the biggest challenges of teaching to teachers who are not the same profession as your future profession? | Write an answer. |
| 7. What are the challenges of pre-clinical teaching? |  |
| 8. What are the advantages of pre-clinical teaching? |  |
