# Supplementary material for: How to create a faculty development program that transforms medical education according to actual institutional needs: evidence-based approach and experience at the University of Rijeka, Faculty of Medicine, Croatia
Source: Front Med (Lausanne). 2025 Feb 18;12:1513119. doi: 10.3389/fmed.2025.1513119 (PMC11876177; doi:10.3389/fmed.2025.1513119)
Supplement: Supplementary file 2 [file Table_2.DOCX]

Supplementary Material 2

Supplementary table 2. The “MPME questionnaire”

| **GROUP 1 - PLANNING OF TEACHING**  Evaluate your level of competency for: | **ANSWER** |
| --- | --- |
| 1. creating a syllabus | Rate on the scale from 1 to 5. |
| 1. creating a lesson plan |  |
| 1. conducting authentical medical education (based on actual needs for the future profession of students) |  |
| 1. knowing and conducting the roles of a course coordinator |  |
| 1. knowing and conducting the roles of a teaching associate |  |
| 1. designing and writing aims of lessons/courses |  |
| 1. designing and writing learning outcomes of lessons/courses |  |
| 1. designing and writing content of lessons/courses |  |
| 1. choosing the form of teaching according to learning outcomes of teaching units |  |
| **GROUP 2 - SELECTING LEARNING METHODS, CREATING TEACHING MATERIALS AND CONDUCTING LESSONS** |  |
| 1. giving feedback to students according to structured models |  |
| 1. establishing interaction with students during classes |  |
| 1. choosing a learning method according to learning outcomes of teaching units |  |
| 1. using authentic methods of passive learning in medical education |  |
| 1. using authentic methods of active learning in medical education |  |
| 1. using presentation skills |  |
| 1. using facilitation skills |  |
| 1. creating own teaching materials |  |
| **GROUP 3 - MONITORING PROGRESS AND ASSESSING STUDENTS AND TEACHING** |  |
| 1. aligning the assessment system with the credit load of a course |  |
| 1. choosing an assessment method according to learning outcomes and methods |  |
| 1. creating different methods of written assessment (e.g. MCQ) |  |
| 1. regular conduction of student evaluation of teaching |  |
| **GROUP 4A - CLINICAL TEACHING** |  |
| 1. teaching clinical reasoning |  |
| 1. orienting students |  |
| 1. using educational strategies for teaching in different clinical environments (e.g. one-minute preceptor) |  |
| 1. teaching clinical skills |  |
| 1. teaching in clinical simulation |  |
| 1. choosing an assessment method for the evaluation of clinical competence according to learning outcomes and learning methods |  |
| **GROUP 4B - PRE-CLINICAL TEACHING** |  |
| 1. teaching clinical reasoning |  |
| 1. orienting students |  |
| 1. applying early integration of clinical content into pre-clinical teaching |  |
| 1. knowing educational strategies for teaching in authentic settings for the future profession of students (e.g. teaching in clinical environment) |  |
| **GROUP 5 - APPLICATION OF E-LEARNING TOOLS IN MEDICAL EDUCATION** |  |
| 1. creating and administrating e-courses in the Moodle LMS |  |
| 1. choosing and creating interactive contents in the Moodle LMS |  |
| 1. monitoring and assessing students' work in virtual environment |  |
