# Supplementary material for: How to create a faculty development program that transforms medical education according to actual institutional needs: evidence-based approach and experience at the University of Rijeka, Faculty of Medicine, Croatia
Source: Front Med (Lausanne). 2025 Feb 18;12:1513119. doi: 10.3389/fmed.2025.1513119 (PMC11876177; doi:10.3389/fmed.2025.1513119)
Supplement: Supplementary file 3 [file Table_3.DOCX]

Supplementary Material 3

Supplementary table 3. List of Meducast and MedXperience innovative video learning formats created from the “Medical students’ focus group” for the MPME FDP (in alphabetical order)

| **LIST OF MEDUCASTS** |
| --- |
| (Non)uniformity of teachers in the same teaching units  Advantages and challenges in conducting oral examinations  Advantages and limitations of lectures  Advantages and limitations of seminars and practicals  Authentical medical education  Characteristics of adults as students  How do students master clinical skills?  How should the forms of teaching differ?  Learning styles  Learning theories in medical education  On the experience of receiving feedback during classes  On the importance of course syllabi  On the importance of vertical and horizontal integration  Principles of learning in adults  Roles of course coordinators  The most common mistakes in written tests  What are and why learning outcomes are important?  What is assessed in medical education?  What should the mandatory literature look like? |
| **LIST OF MEDXPERIENCES** |
| (Non)uniformity of teachers in the same teaching units  Authenticity of medical education  On the importance of vertical and horizontal integration  Peer teaching at the Department of Anesthesiology, Reanimatology, Emergency and Intensive Care Medicine  Presentation skills |
